# Quantum phase transitions in nonhermitian harmonic oscillator

**DOI:** 10.1038/s41598-020-75468-w

**Published:** 2020-10-28

**Authors:** Miloslav Znojil

**Affiliations:** 1 Department of Physics, Faculty of Science, University of Hradec Králové, Rokitanského 62, 50003 Hradec Králové, Czech Republic; 2 The Czech Academy of Sciences, Nuclear Physics Institute, Hlavní 130, 250 68 Řež, Czech Republic

**Keywords:** Mathematics and computing, Nanoscience and technology, Optics and photonics, Physics

## Abstract

The Stone theorem requires that in a physical Hilbert space ${{{\mathcal {H}}}}$ the time-evolution of a stable quantum system is unitary if and only if the corresponding Hamiltonian *H* is self-adjoint. Sometimes, a simpler picture of the evolution may be constructed in a manifestly unphysical Hilbert space ${{{\mathcal {K}}}}$ in which *H* is nonhermitian but ${{\mathcal {PT}}}$-symmetric. In applications, unfortunately, one only rarely succeeds in circumventing the key technical obstacle which lies in the necessary reconstruction of the physical Hilbert space ${{{\mathcal {H}}}}$. For a ${{\mathcal {PT}}}$-symmetric version of the spiked harmonic oscillator we show that in the dynamical regime of the unavoided level crossings such a reconstruction of ${{{\mathcal {H}}}}$ becomes feasible and, moreover, obtainable by non-numerical means. The general form of such a reconstruction of ${{{\mathcal {H}}}}$ enables one to render every exceptional unavoided-crossing point tractable as a genuine, phenomenologically most appealing quantum-phase-transition instant.

## Introduction

In the Carroll’s book about Alice’s adventures one reads that the “Cheshire Cat appears in a tree”, and then he “disappears but his grin remains behind to float on its own in the air”^1^. In a fairly close parallel to the Carroll’s story (and to its occasional, time-to-time use in physics^2,3^), the appearance, more than twenty years ago^4,5^, of the imaginary cubic potential $V(x)={\mathrm{i}}x^3$ resembled the Cheshire Cat of quantum theory. The study of the model directed the community of quantum physicists towards the wide acceptance of the concept of parity-times-time-reversal symmetry (${{\mathcal {PT}}}$-symmetry) in quantum mechanics of unitary systems^6^. In this new formulation of the theory a simpler picture of the evolution may sometimes be constructed^7^.

In a continued parallel, “the grin” (i.e., the inspiring idea of ${{\mathcal {PT}}}$-symmetry) is still “floating in the air” at present^8,9^. At the same time, the imaginary cubic potential itself has repeatedly been shown to disappear from the scene of physics because “there is no quantum-mechanical Hamiltonian associated with it”^10^. An evidence is now available that many nonhermitian, Cheshire-Cat-resembling models of dynamics exhibit certain “unexpected wild properties”^11^. Many people now believe that at least some of the similar benchmark models “are not equivalent to Hermitian models, but that they rather form a separate model class with purely real spectra”^12^.

In our present paper we intend to weaken such a wave of scepticism. We will show that there exist non-trivial nonhermitian quantum systems living in infinite-dimensional Hilbert spaces in which, after an appropriate formulation of the theory, even an innovative, quantum-phase-transition-opening “wild” behavior can still be given an entirely conventional, unitary-evolution interpretation and explanation compatible with the dictum of standard textbooks.

The purpose will be served by the nonhermitian but ${{\mathcal {PT}}}$-symmetric toy model (with the real spectrum) represented by the ordinary differential Schrödinger equation

1 $$\begin{aligned} \left( -\,\frac{d^2}{dr^2} + \frac{G}{r^2}+r^2 \right) \, \varphi _{}(r) = E_{} \, \varphi _{}(r)\,. \end{aligned}$$

In conventional textbooks^13^ the model is used to describe the radial motion of a particle in a *D*-dimensional harmonic oscillator well $V(\vec {r})=|\vec {r}|^2$. In these textbooks the authors also add a comment that even when $G > -1/4$ is negative, the quantum system remains stable, in a remarkable contrast to its unstable classical analogue.

The latter remarks do not exhaust the list of the remarkable features of model (1). In 1999, in a way inspired by Bender and Boettcher^4^ we showed, in Ref.^14^, that under the same constraint $G > -1/4$ the model remains stable even when it ceases to be Hermitian. We proved, in particular, that the complex shift of the line of coordinates

2 $$\begin{aligned} r\ \rightarrow \ r(x)=x-{\mathrm{i}} c \ \in \ {\mathbb {C}}\,, \quad c>0\,,\ \ \ x \in (-\infty ,\infty )\, \end{aligned}$$

in the same ordinary differential equation (1) still keeps the energy spectrum real, discrete and bounded from below. The regularization of the centrifugal-type singularity in the resulting nonhermitian but ${{\mathcal {PT}}}$-symmetric (i.e., parity-times-time-reversal-symmetric^6,15^) Hamiltonian

3 $$\begin{aligned} H^{(\alpha )}= -\,\frac{d^2}{dx^2} + (x-ic)^2 + \frac{G}{(x-ic)^2}\,, \ \ \ \ \alpha =\sqrt{G+1/4}>0\,, \ \ \ \ c>0\,, \ \ \ \ x \in (-\infty ,\infty ) \end{aligned}$$

made the model eligible as an unusual but still exactly solvable example in supersymmetric quantum mechanics^16^.

Mathematically, our Hamiltonian operator $H^{(\alpha )}$ is defined in Hilbert space ${{{\mathcal {K}}}}=L^2({\mathbb {R}})$ of square-integrable functions of the new real variable *x*. In Ref.^14^ it has been shown that in spite of the manifest nonhermiticity of Hamiltonian (3) in ${{{\mathcal {K}}}}$, its spectrum is all real and defined, in terms of two quantum numbers, by compact formula

4 $$\begin{aligned} E=E_{n}^{(Q)}=4n+2 - 2 Q \alpha \,, \quad Q = \pm 1\,, \quad n = 0, 1, 2, \ldots \,. \end{aligned}$$

**Figure 1:**
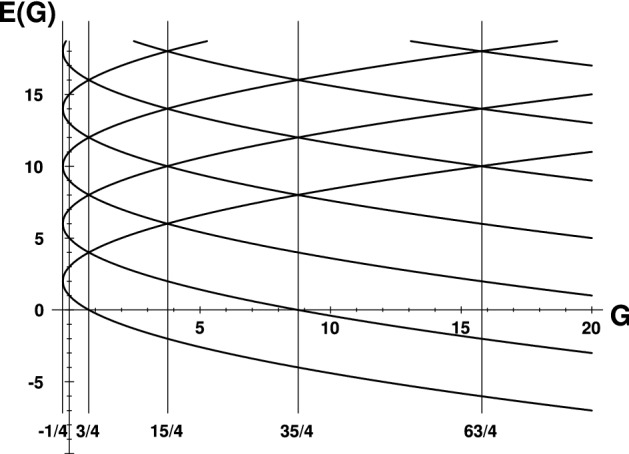
*G*-dependence of spectrum of ${{\mathcal {PT}}}$-symmetric harmonic oscillator (3). Vertical lines mark the exceptional-point values of coupling $G^{(EP)}_\alpha =\alpha ^2-1/4$ at $\alpha =\alpha ^{(EP)}=0,1,2,3$ and 4.

As functions of the coupling *G* of the regularized centrifugal-like spike these eigenvalues are sampled in Fig. 1.

In our present paper a long missing constructive probabilistic interpretation of such an exactly solvable quantum model will be presented in a restriction to the most interesting dynamical regimes which are not too far from the instants of phase transitions called exceptional points (EPs,^17^).

## Physics behind ${{\mathcal {PT}}}$-symmetric harmonic oscillator

At the time of the publication of Ref.^14^ in 1999, a consistent physical unitary-evolution interpretation of similar nonhermitian Hamiltonians has not been available yet. During the first years of the new millennium people still preferred the conventional phenomenological treatment of similar models, based on the widely known Feshbach’s effective-Hamiltonian philosophy^18,19^. Only step by step it has been clarified that one has to distinguish, very strictly, between such a traditional, “manifestly nonhermitian” approach (in which the systems are, in general, resonant or dissipative, and in which the phenomenologically meaningful spectra of energies may be, and are, in general, complex) and its “hiddenly Hermitian” alternative as proposed by Bender with Boettcher (in^4^ they insisted on the strict reality and stable bound-state physical meaning of the energy spectra).

In our present paper we will only pay attention to the latter branch of the theory. Readers interested in the current status of the former, wider branch of research may find its representative sample, e.g., in the very recent edited book^8^. In contrast, several extensive introductions into the latter, unitary quantum theory using nonhermitian Hamiltonians with real spectra may be found reviewed, e.g., in the pair of books^9,20^.

In an application of the latter, unitary-evolution approach to our present model (3) it is necessary to point out, first of all, that the underlying, user-friendly Hilbert space ${{{\mathcal {K}}}}=L^2({\mathbb {R}})$ loses the status of the physical space of states with the conventional probabilistic interpretation. For this reason, one has to emphasize that also the variable *x* still *cannot * carry the physical meaning of an observable of a particle position^21^. In this setting, the intuitively appealing concept of $\,{{\mathcal {PT}}}$-symmetry must be assigned its amended mathematical meaning of a Krein-space-based ${{{\mathcal {P}}}}$-pseudo-Hermiticity of the Hamiltonian^7,22,23^.

In a retrospective it is possible to say that several years were needed for an ultimate correct reformulation of quantum mechanics in which one comes to the conclusion that even the manifestly nonhermitian Hamitonians with real spectra may still generate a stable and unitary evolution. Incidentally, during the step-by-step discoveries of the ultimate consistent formulation of the theory (see, e.g.,^6, 7^) people were also rediscovering the applicability of an older knowledge of the problem not only in abstract mathematics^24^ but also in several pragmatic reinterpretations of the first principles of quantum mechanics by physicists^25,26^.

### Three Hilbert space formulation of quantum mechanics

During the process of understanding of the Bender’s and Boettcher’s conjectures^4^ it appeared necessary to replace, first of all, the mathematically friendly Hilbert space ${{{\mathcal {K}}}}$ by its unitarity-compatible alternative (in a way recommended in^27^ we will denote it by dedicated symbol ${{{\mathcal {H}}}}$ in what follows). These two Hilbert spaces differ just by na amendment of the inner product (·,·)^26^. In the standard Dirac’s bra-ket notation we may write

5 $$\begin{aligned} (\psi _a,\psi _b)_{{{\mathcal {K}}}}=\langle \psi _a|\psi _b \rangle \,, \quad (\psi _a,\psi _b)_{{{\mathcal {H}}}}=\langle \psi _a|\Theta |\psi _b \rangle \,. \end{aligned}$$

Here, the *ad hoc,*, Hilbert-space-metric operator $\Theta$ must satisfy several compatibility conditions, thoroughly studied and listed in^26^. For our present purposes we only recall that this operator must be self-adjoint in ${{{\mathcal {K}}}}$, $\Theta =\Theta ^\dagger$. This, as a consequence, guarantees the unitarity of the evolution of the system in ${{{\mathcal {H}}}}$. Moreover, one must also guarantee that this operator is bounded and invertible, with bounded inverse^26^. Last but not least, we need that the use of this Hilbert-space-metric operator reinstalls the correct probabilistic contents of the underlying quantum theory, i.e., that the condition $\Theta >0$ of its positive definiteness leads to the standard norm in ${{{\mathcal {H}}}}$.

This means that the evolution generated by *H* will be unitary in ${{{\mathcal {H}}}}$^28,29^. At the same time, this implies that the metric must be, by construction, Hamiltonian-dependent, i.e., such that

6 $$\begin{aligned} H^\dagger \,\Theta =\Theta \,H\,. \end{aligned}$$

Fortunately, once we factorize the metric

7 $$\begin{aligned} \Theta =\Omega ^\dagger \Omega \end{aligned}$$

we reveal that all of the above requirements are compatible, and that the operator $\Omega$ maps the ket-vector elements $|\psi \rangle$ of ${{{\mathcal {K}}}}$ (or, equivalently, of ${{{\mathcal {H}}}}$) on the new, “curly” kets which span another, third Hilbert space denoted as ${{{\mathcal {L}}}}$,

8 $$\begin{aligned} |\psi \succ \,\,= \Omega \,|\psi \rangle \,, \quad |\psi \rangle \, \in \,{{{\mathcal {H}}}}\,, \quad |\psi \succ \,\,\, \in \,{{{\mathcal {L}}}}\,. \end{aligned}$$

This construction implies the equivalence between the following two inner products,

9 $$\begin{aligned} (\psi _a,\psi _b)_{{{\mathcal {H}}}}=(\psi _a,\psi _b)_{{{\mathcal {L}}}}\,. \end{aligned}$$

We may conclude that the quantum system in question may be represented by the Hamiltonian *H* acting in Hilbert space ${{{\mathcal {H}}}}$ or, equivalently, by the Hamiltonian

10 $$\begin{aligned} {\mathfrak {h}}=\Omega \,H\,\Omega ^{-1} \end{aligned}$$

defined in Hilbert space ${{{\mathcal {L}}}}$. In this framework we may say that ${\mathfrak {h}}$ is self-adjoint in ${{{\mathcal {L}}}}$ while *H* is self-adjoint in ${{{\mathcal {H}}}}$^29^. The third, manifestly unphysical Hilbert space ${{{\mathcal {K}}}}$ is just a mathematically preferred auxiliary space in which the calculations are all performed—this is the reason why *H* is often (and misleadingly) called nonhermitian.

In an application of the three-Hilbert-space picture to our harmonic oscillator Hamiltonian we may also observe that it is nonhermitian in the manifestly unphysical Hilbert space ${{{\mathcal {K}}}}=L^2({\mathbb {R}})$. Obviously, unless we specify the physical Hilbert space ${{{\mathcal {H}}}}$ [i.e., the metric $\Theta$], the description of the system remains unfinished, leaving the information about physics *incomplete*. The necessity of the completion (i.e., of the specification of metric $\Theta$) follows from the necessity of the standard probabilistic interpretation of the model. In applications, such a requirement reflects the weakest point of the whole theory. In fact, for a long time it remained unnoticed that the present spiked harmonic oscillator model offers one of the rare opportunities of its consequent and complete implementation.

### Exceptional points

Besides an expected confirmation of complexification of the whole spectrum of model (3) at negative $\alpha <0$ (the effect widely known under the nickname of a spontaneous breakdown of ${{\mathcal {PT}}}$-symmetry^4,30^), one of the key results of paper^14^ was the observation that at the positive integer values of $\alpha =1,2,\ldots$ the energy levels cross but remain real. Due to the exact solvability of the model it was easy to reveal that at all of these values of the parameter marking the unavoided eigenvalue crossings were accompanied by the parallelization and degeneracy of the related pairs of eigenvectors. Indeed, for the bound state wave functions expressed in terms of Laguerre polynomials,

11 $$\begin{aligned} \varphi (x) = const. \,(x-ic)^{-Q \alpha +1/2}e^{-(x-ic)^2/2} \ L^{(-Q \alpha )}_n \left[ (x-ic)^2 \right] \,,\quad n=0,1,\ldots \end{aligned}$$

the rigorous proof of the parallelizations was based on the elementary identities like

$$\begin{aligned} L^{(-1)}_{n+1}\left[ (x-ic)^2 \right] =-(x-ic)^2\,L^{(1)}_n\left[ (x-ic)^2 \right] \end{aligned}$$

etc. Using the terminology as introduced by Kato^17^ all of the integer values of $\alpha =0,1,\ldots$ may be called exceptional points (EPs). At these values, operator $H^{(\alpha )}$ ceases to be diagonalizable. In the context of quantum mechanics this has the following important consequence (see the reasons, e.g., in^7^).

#### **Lemma 1**

^14^ *Operator* (3) *may play the role of Hamiltonian of a unitary quantum system only if* $\alpha >0$ *and* $\alpha \notin {\mathbb {Z}}$.

**Table 1:** EP degeneracies.

$\alpha =$	0	1	2	3	4
${{E_n^{(Q)}}(G)}$	$(G=-{1}/{4})$	$(G={3}/{4})$	$(G={15}/{4})$	$(G={35}/{4})$	$(G={63}/{4})$
$$\vdots$$					
$-$6					$E_0^{(+)}$
$-$4				$E_0^{(+)}$	
$-$2			$E_0^{(+)}$		$E_1^{(+)}$
0		$E_0^{(+)}$		$E_1^{(+)}$	
2	$E_0^{(+)}=E_0^{(-)}$		$E_1^{(+)}$		$E_2^{(+)}$
4		$E_0^{(-)}=E_1^{(+)}$		$E_2^{(+)}$	
6	$E_1^{(+)}=E_1^{(-)}$		$E_0^{(-)}=E_2^{(+)}$		$E_3^{(+)}$
8		$E_1^{(-)}=E_2^{(+)}$		$E_0^{(-)}=E_3^{(+)}$	
10	$E_2^{(+)}=E_2^{(-)}$	$\vdots$	$E_1^{(-)}=E_3^{(+)}$	$\vdots$	$E_1^{(-)}=E_4^{(+)}$
$\vdots$	$\vdots$		$\vdots$		$\vdots$

The detailed nature of EP-related degeneracies can vary with our choice of $\alpha ^{(EP)}=\alpha ^{(EP)}_K=K$ where $K =0,1,\ldots$ (see Table 1). At these points the lost possibility of diagonalization of $H^{(\alpha )}$ can only be replaced by its canonical representation,

12 $$\begin{aligned} H^{{(\alpha )}}_{}\, Q^{{(\alpha )}}_{} = Q^{{(\alpha )}}_{}\,{{{\mathcal {J}}}}^{{(\alpha )}} \,,\quad \alpha =\alpha ^{{(EP)}}\in {\mathbb {Z}}\,. \end{aligned}$$

An optimal choice of the infinite-dimensional canonical representative ${{{\mathcal {J}}}}^{{(\alpha )}}$ of the EP limit of the Hamiltonian will be specified below. This choice will enable us to treat the transition-matrix solutions $Q^{{(\alpha )}}_{}$ of Eq. (12) as a certain degenerate EP analogue of the set of eigenvectors forming an unperturbed basis. In such a perspective our recent experience with the EP-based perturbation theory will find its new application as a tool of making, finally, the consistent and constructive physical interpretation of our nonhermitian but unitary harmonic oscillator quantum model near its EP singularities complete.

Expectedly^26^, without an additional information about dynamics there will be infinitely many such completions. In the related literature, unfortunately, one rarely finds a sufficiently nontrivial example of such a variability of options. In our present paper such an example is provided.

## Results

### Physical Hilbert space of oscillator near the spontaneous breakdown of ${{\mathcal {PT}}}$-symmetry ($\alpha ^{(EP)}=0$)

Let us initiate our analysis of oscillator (3) in the dynamical regime of the smallest positive parameters $\alpha$. Only in the next section we will make the analysis complete by extending it to all of the EP neighborhoods of $\alpha \approx K$ with $K=1,2, \ldots$.

Near the lowermost EP limit $\alpha \rightarrow 0^+$ an inspection of Fig. 1 reveals that the full, infinite-dimensional Hilbert space may be decomposed into a sequence of two-dimensional subspaces ${{{\mathcal {K}}}}^{[2]}_{(n)}$,

13 $$\begin{aligned} {{{\mathcal {K}}}}=\bigoplus _{n=0}^\infty \,{{{\mathcal {K}}}}^{[2]}_{(n)}\,. \end{aligned}$$

The vanishing$-\alpha$ loss of the diagonalizability of $H^{(\alpha )}$ may be best reflected by the choice of the canonical representation matrix ${{{\mathcal {J}}}}^{{(0)}}$ of Eq. (12) in the following block-diagonal-matrix form of a direct sum of Jordan matrices,

14 $$\begin{aligned} {{{\mathcal {J}}}}^{{(0)}}=J^{[2]}(2)\bigoplus J^{[2]}(6)\bigoplus J^{[2]}(10) \bigoplus \ldots \,, \quad J^{[2]}(E)= \left( \begin{array}{cc} E&{}\quad 1\\ 0&{}\quad E \end{array} \right) . \end{aligned}$$

Having specified this matrix we have to solve Eq. (12) yielding the infinite-dimensional transition matrix. The columns of this matrix may then play the role of an unperturbed basis in ${{{\mathcal {K}}}}$. Such a construction generates, finally, a simplified isospectral zero-order representation of our Hamiltonian,

15 $$\begin{aligned} {\mathfrak {H}}^{(0)}(\alpha )= \left[ Q^{{(0)}}_{} \right] ^{-1}\,H^{{(\alpha )}}_{}\, Q^{{(0)}}_{}= {{{\mathcal {J}}}}^{{(0)}} + {\mathrm{corrections}} \,,\quad 0<\alpha \ll 1\,. \end{aligned}$$

In other words this means that our Hamiltonian will have the infinite-dimensional block-diagonal matrix structure,

$$\begin{aligned} {\mathfrak {H}}^{(0)}(\alpha )= \left( \begin{array}{cc|cc|cc} 2&{}1&{}0&{}0&{}0&{}\ldots \\ 0&{}2&{}0&{}0&{}0&{}\ldots \\ \hline 0&{}0&{}6&{}1&{}0&{}\ldots \\ 0&{}0&{}0&{}6&{}0&{}\ldots \\ \hline 0&{}0&{}0&{}0&{}10&{}\ldots \\ \vdots &{}\vdots &{}\vdots &{}\vdots &{}\ddots &{}\ddots \end{array} \right) \ +\ {\mathrm{corrections}} \end{aligned}$$

where the blocks are the two-by-two Jordan matrices. It is now necessary to specify the first-order correction term in (15).

In the dynamical regime of small and positive $\alpha$s, *i.e.*, close to the leftmost EP instability at $\alpha ^{(EP)}_0 = 0$ we have to describe the energies as functions of the coupling constant *G*. Indeed, once we set $G=G^{(EP)}+ \xi$ and once we rewrite formula (4) for energies as a function of $\xi$, we notice the qualitative difference between the left and right vicinities of $\xi =0$. As long as only the right, real-energy vicinity with $\xi >0$ in $E_n^{(\pm )}= 4n+2 \pm 2\,\sqrt{\xi }$ is of our present interest, we know that at its EP boundary the whole spectrum degenerates pairwise, $\lim _{\alpha \rightarrow 0}\,E^{(\pm )}_{{n}}\rightarrow 4n+2$, $n=0,1,\ldots$.

Our recent experience with corrections to non-diagonalizable matrices^31^ warns us against a naive expectation that the correction term in (15) should be of order ${{{\mathcal {O}}}}(\alpha )$. An independent version of the same warning came also from Ref.^32^ and/or from inspection of Fig. 1. We found that the dominant, leading-order correction appearing in Eq. (15) may be written in an apparently counterintuitive but still remarkably elementary explicit form,

16 $$\begin{aligned} {\mathfrak {H}}^{(0)}(\alpha )= {{{\mathcal {J}}}}^{{(0)}} + \xi {{{\mathcal {V}}}}^{{(0)}} + {\text {higher order corrections}} \,,\quad \xi ={{{\mathcal {O}}}}(\alpha ^2) \end{aligned}$$

with elementary block-diagonal matrix of perturbations

17 $$\begin{aligned} {{{\mathcal {V}}}}^{{(0)}}= \left[ J^{[2]}(0) \right] ^T\,\bigoplus \left[ J^{[2]}(0) \right] ^T\,\bigoplus \left[ J^{[2]}(0) \right] ^T\,\bigoplus \ldots \end{aligned}$$

where, the superscript $^T$ marks the matrix transposition.

The main consequence of these formulae is that in every two-dimensional subspace ${{{\mathcal {K}}}}^{[2]}_{(n)}$ we have a block-diagonalized leading-order Hamiltonian

18 $$\begin{aligned} {\mathfrak {H}}^{(0)}(\alpha ) \approx {\mathfrak {H}}^{(0)}_0(\alpha ) = {H}^{[2]}_{(0)}(\xi ) \,\bigoplus \, {H}^{[2]}_{(1)}(\xi ) \,\bigoplus \,\ldots \end{aligned}$$

where

19 $$\begin{aligned} {H}^{[2]}_{(n)}(\xi )= J^{[2]}(E_n^{(+)})+\xi \, \left[ J^{[2]}(0) \right] ^T= \left( \begin{array}{cc} E_n^{(+)}&{}1\\ \xi &{}E_n^{(+)} \end{array} \right) \,,\quad E_n^{(+)}=\left. E_n^{(+)}\right| _{\alpha =0} =4n+2\,. \end{aligned}$$

For the latter submatrices we can solve the related time-independent Schrödinger equations in closed form,

20 $$\begin{aligned} H^{[2]}_{(n)}(\xi )\, \left( \begin{array}{c} 1\\ \eta _\pm \end{array} \right) = \left( E_n^{(+)}+\eta _\pm \right) \, \left( \begin{array}{c} 1\\ \eta _\pm \end{array} \right) \,, \quad \eta _\pm =\pm \sqrt{\xi }\,. \end{aligned}$$

This has the following consequence.

#### **Lemma 2**

*For approximate two by two matrix Hamiltonians* (19) *the unfolding energies are real if and only if the small parameter* $\xi$ *is non-negative,* $\xi \ge 0$.

At non-negative $\xi$ we have $\xi =\alpha ^2$ and $\eta _\pm =\pm \alpha$ in (20). The approximate Hamiltonian (19) may be then made Hermitian along the lines outlined above. Via a mere redefinition of inner products (5) our unphysical but mathematically optimal Hilbert space ${{{\mathcal {K}}}}^{[2]}_{(n)}$ is converted into its correct physical alternative ${{{\mathcal {H}}}}^{[2]}_{(n)}$.

#### **Lemma 3**

*Metric operators* $\Theta$ *making Hamiltonian* (19) *Hermitian (in* ${{{\mathcal {H}}}}^{[2]}_{(n)}$*) read*

21 $$\begin{aligned} \Theta =\Theta ^{[2]}_{(n)}(\alpha ,b_n)= \left( \begin{array}{cc} \alpha &{}b_n\\ b_n&{}1/\alpha \end{array} \right) \, \end{aligned}$$

*and form a one-parametric family numbered by a real variable* $b_n$ *such that* $|b_n|<1$.

#### *Proof*

The Hermiticity of matrix $H^{[2]}_{(n)}(\xi )$ in the physical Hilbert space ${{{\mathcal {H}}}}^{[2]}_{(n)}$ means that this matrix satisfies condition (6). This condition (written in ${{{\mathcal {K}}}}^{[2]}_{(n)}$) may be perceived as a set of linear equations for the matrix elements of the unknown matrix $\Theta$. This matrix must be Hermitian and positive definite^26^. Under these constraints, an easy algebra leads to the result. $\square$

#### **Theorem 4**

*At small* $\alpha$ *the infinite-dimensional matrix Hamiltonian* ${\mathfrak {H}}^{(0)}_0(\alpha )$ *of Eq.* (18) *becomes Hermitian in the* *ad hoc*  *physical Hilbert space*

22 $$\begin{aligned} {{{\mathcal {H}}}}=\bigoplus _{n=0}^\infty \,{{{\mathcal {H}}}}^{[2]}_{(n)} \end{aligned}$$

*whenever we introduce, in* ${{{\mathcal {K}}}}=\bigoplus _n\,{{{\mathcal {K}}}}^{[2]}_{(n)}$*, one of the amended, nontrivial inner-product metrics*

23 $$\begin{aligned} \Theta =\bigoplus _{n=0}^\infty \,\Theta ^{[2]}_{(n)}(\alpha ,b_n)\,. \end{aligned}$$

*The optional sequence of parameters* $b_n \in (-1,1)$ *with* $n=0,1,\ldots$ *is arbitrary.*

#### *Proof*

The infinite-dimensional matrix Hamiltonian (16) must be shown compatible with the Dieudonné’s Hermiticity condition (6), but this follows from the block-diagonality of the participating infinite-dimensional matrices, and from Lemma 3. $\square$

We see that at sufficiently small parameters $\alpha >0$, our ${{\mathcal {PT}}}$-symmetric harmonic-oscillator Hamiltonian (3) defined in auxiliary, unphysical Hilbert space ${{{\mathcal {K}}}} = L_2(-\infty ,\infty )$ of Eq. (13) acquires the status of standard self-adjoint generator of unitary evolution. Nevertheless, different choices of the sequence of parameters $\{b_n\}$ define phenomenologically non-equivalent quantum systems. In any such a system the observables must be represented by operators $\Lambda$ which are self-adjoint in the physical Hilbert space ${{{\mathcal {H}}}}$ of Eq. (22). Even for the block-diagonal subset $\Lambda =\bigoplus _n \Lambda ^{[2]}_{(n)}$ of observables the general form of their admissible submatrices

24 $$\begin{aligned} \Lambda ^{[2]}_{(n)}= \left( \begin{array}{cc} u &{}\quad v\\ y&{}\quad z \end{array} \right) \, \end{aligned}$$

remains $b_n$-dependent in general. Indeed, in a parallel to Eq. (6) these submatrices must satisfy the metric-dependent Hermiticity constraint

25 $$\begin{aligned} \left[ \Lambda ^{[2]}_{(n)}\right] ^\dagger \, \Theta ^{[2]}_{(n)}(\alpha ,b_n)= \Theta ^{[2]}_{(n)}(\alpha ,b_n)\, \Lambda ^{[2]}_{(n)}\,. \end{aligned}$$

#### **Lemma 5**

*Condition* (25) *is satisfied if and only if we restrict*

26 $$\begin{aligned} y=y(b_n)= \alpha ^2v+\alpha \,b_n\,(z-u) \, \end{aligned}$$

*in* (24).

In an elementary check, the latter construction of observables reproduces the initial leading-order Hamiltonian at $v=1$ and $u=z=0$. It is also easy to verify that the most popular complementary observable of charge^6^ is obtained at $v=1/\alpha$ and $u=z=b_n=0$.

Marginally, let us add that once we reparametrize $\alpha =\exp t$ and $b_n=\cos \phi$ in (21) and once we put $\phi =\mu +\nu$ we may also factorize the metric (cf. Eq. (7)) yielding

27 $$\begin{aligned} \Omega ^{[2]}_{(n)}= \left( \begin{array}{cc} p&{}a\\ a&{}q \end{array} \right) \,,\quad p=e^{t/2} \sin \mu \,, \quad q=e^{-t/2} \sin \nu \,, \quad a=e^{t/2} \cos \mu =e^{-t/2} \cos \nu \,. \end{aligned}$$

On these grounds, whenever needed, we may perform transition to the third Hilbert space ${{{\mathcal {L}}}}$ using Eq. (8). Redundant as this step may seem to be, the work in the latter space is often recommended in conventional textbooks, mainly for establishing easier contacts with experimentalists (cf., e.g.,^21,33^).

### Physical Hilbert spaces of oscillators near unavoided level crossings ($\alpha ^{(EP)}=1,2,\ldots$)

In Fig. 1 we notice a significant qualitative difference between the leftmost EP at $\alpha =\alpha ^{(EP)}_0 = 0$ (to the left of which the spectrum complexifies) and the remaining EP family of $\alpha ^{(EP)}_K= K$ with $K=1,2,\ldots$ (in the respective vicinities of which the spectra remain real). In what follows we intend to show that at $K \ge 1$ such a qualitative phenomenological difference is also reflected by the related mathematics.

First of all we notice that in the limit $\alpha \rightarrow \alpha ^{(EP)}_K$ the *K*-plet of the lowermost energy levels remains non-degenerate (ND). In a small vicinity of $\alpha ^{(EP)}_K$ the *K*-dimensional Hilbert space ${{{\mathcal {K}}}}^{[K]}_{ND}$ spanned by the corresponding wave functions may be characterized, for this reason, by the unit-matrix metric of textbooks, $\Theta ^{[K]}_{ND}=I$. Hence, this subspace may be treated as equivalent to its two physical alternatives, ${{{\mathcal {K}}}}^{[K]}_{ND}\equiv {{{\mathcal {H}}}}^{[K]}_{ND}\equiv {{{\mathcal {H}}}}^{[K]}_{ND}$. For this reason the full Hilbert spaces

28 $$\begin{aligned} {{{\mathcal {K}}}}= {{{\mathcal {K}}}}^{[K]}_{ND}\, \bigoplus \,{{{\mathcal {K}}}}^{[2]}_{(0)} \bigoplus \,{{{\mathcal {K}}}}^{[2]}_{(1)} \bigoplus \,\ldots \, ,\quad {{{\mathcal {H}}}}= {{{\mathcal {H}}}}^{[K]}_{ND}\, \bigoplus \,{{{\mathcal {H}}}}^{[2]}_{(0)} \bigoplus \,{{{\mathcal {H}}}}^{[2]}_{(1)} \bigoplus \,\ldots \, \end{aligned}$$

may be, for our present purpose of the construction of its physics-representing amendment, reduced to the respective relevant tilded subspaces

29 $$\begin{aligned} \widetilde{{{\mathcal {K}}}}=\widetilde{{{\mathcal {K}}}}^{[2]}_{(0)} \bigoplus \,\widetilde{{{\mathcal {K}}}}^{[2]}_{(1)} \bigoplus \,\widetilde{{{\mathcal {K}}}}^{[2]}_{(2)}\,\bigoplus \,\ldots \,, \quad \widetilde{{{\mathcal {H}}}}=\widetilde{{{\mathcal {H}}}}^{[2]}_{(0)} \bigoplus \,\widetilde{{{\mathcal {H}}}}^{[2]}_{(1)} \bigoplus \,\widetilde{{{\mathcal {H}}}}^{[2]}_{(2)}\,\bigoplus \,\ldots \, . \end{aligned}$$

In the small left and right vicinities of exceptional points $\alpha ^{(EP)} =K\ge 1$ the reduction of attention will also involve the omission, from our considerations, of the trivial, diagonal-matrix sub-Hamiltonian $H^{[K]}_{ND}$ such that

$$\begin{aligned} {\left( H^{[K]}_{ND} \right) }_{jj}=E^{(+)}_j=-2K+2+4j\,, \quad j=0,1,\ldots ,K-1\,. \end{aligned}$$

In the only relevant (i.e., in our notation, in the tilded) part (29) of full spaces the rest of the spectrum remains doubly degenerate forming the sequence sampled in Table 1,

$$\begin{aligned} E^{(-)}_n=E^{(+)}_{n+K}=2K+2+4n\,, \quad n=0,1,\ldots \,. \end{aligned}$$

This enables us to establish, in three steps, several $K>0$ parallels with the preceding $K=0$ results. In the first step we introduce the tilded version of the canonical EP Hamiltonian,

30 $$\begin{aligned} \widetilde{{{\mathcal {J}}}}^{{(K)}}=J^{[2]}(2K+2)\bigoplus J^{[2]}(2K+6) \bigoplus \ldots \,. \end{aligned}$$

Up to the omission of the first *K* non-degenerate levels this is a perfect $K>0$ analogue of the $K=0$ EP Hamiltonian of Eq. (14). In the second step we define the infinite-dimensional tilded transition matrices ${\widetilde{Q}}^{(K)}$ as solutions of a tilded version of Eq. (12). In the third step, as above, we finally use these transition matrices to define the unperturbed basis (cf.^31^).

In the vicinity of $\alpha ^{(EP)}_K=K$, as a result, the tilded $K>0$ analogue of the simplified Hamiltonian of Eq. (16) is obtained,

31 $$\begin{aligned} \widetilde{{\mathfrak {H}}}^{(K)}(\alpha )= \left[ {\widetilde{Q}}^{{(K)}}_{} \right] ^{-1}\,{\widetilde{H}}^{{(\alpha )}}_{}\, {\widetilde{Q}}^{{(K)}}_{}= \widetilde{{{\mathcal {J}}}}^{{(K)}} + \delta ^2 \widetilde{{{\mathcal {V}}}}^{{(K)}} + {\text {higher order corrections}}\,. \end{aligned}$$

The matrix of perturbations itself remains the same as above, $\widetilde{{{\mathcal {V}}}}^{{(K)}}={{{\mathcal {V}}}}^{{(0)}}$ [cf. Eq. (17) above]. What is, nevertheless, different is the role of the new small parameter $\delta =\delta (\alpha )=\alpha -K$. One of the reasons is that the unfolded spectrum remains real at both of its signs. Hence, the approximate leading-order tilded Hamiltonian

32 $$\begin{aligned} \widetilde{{\mathfrak {H}}}^{(K)}_0(\alpha ) = {\widetilde{H}}^{[2]}_{(0)}[\delta (\alpha )] \,\bigoplus \, {\widetilde{H}}^{[2]}_{(1)}[\delta (\alpha )] \,\bigoplus \,\ldots \end{aligned}$$

with

33 $$\begin{aligned} {\widetilde{H}}^{[2]}_{(n)}(\delta )= J^{[2]}(E_n^{(-)})+\delta ^2\, \left[ J^{[2]}(0) \right] ^T= \left( \begin{array}{cc} E_n^{(-)}&{}1\\ \delta ^2 &{}E_n^{(-)} \end{array} \right) \,,\ \ \ \ \ \ \ \ \ \ \ \ \ E_n^{(-)}=\left. E_n^{(-)}\right| _{\alpha =K} =2K+4n+2\, \end{aligned}$$

has different spectral properties determined by the related Schrödinger equation

34 $$\begin{aligned} {\widetilde{H}}^{[2]}_{(n)}(\delta )\, \left( \begin{array}{c} 1\\ \pm \delta \end{array} \right) = \left( E_n^{(+)}\pm \delta \right) \, \left( \begin{array}{c} 1\\ \pm \delta \end{array} \right) \,. \end{aligned}$$

Still, many of the consequences remain similar.

#### **Lemma 6**

*Metric operators making Hamiltonian* (33) *Hermitian in* $\widetilde{{{\mathcal {H}}}}^{[2]}_{(n)}$ *form a one-parametric family*

35 $$\begin{aligned} {\widetilde{\Theta }}^{[2]}_{(n)}(\delta ,c_n)= \left( \begin{array}{cc} \delta &{}\quad c_n\\ c_n&{}\quad 1/\delta \end{array} \right) \, \end{aligned}$$

*where* $\delta =\delta (\alpha )=\alpha -K \ne 0$ *is small, and where* $-1<c_n<1$.

#### *Proof*

The construction is analogous to the one described in the proof of Lemma 3. $\square$

#### **Theorem 7**

*Tilded Hamiltonian* $\widetilde{{\mathfrak {H}}}^{(K)}_0(\alpha )$ *of Eq.* (32) *is Hermitian in any tilded physical Hilbert space* $\widetilde{{{\mathcal {H}}}}$ *of Eq.* (29) *characterized by the metric*

36 $$\begin{aligned} {\widetilde{\Theta }}=\bigoplus _{n=0}^\infty \, {\widetilde{\Theta }}^{[2]}_{(n)}[\delta (\alpha ),c_n)\, \end{aligned}$$

*where all of the parameters* $c_n \in (-1,1)$ *are variable.*

#### *Proof*

In comparison with Theorem 4 the only modification of the proof is that now we ignore the low-lying bound-state *K*-plets as controlled by trivial metric $\Theta ^{[K]}_{ND}=I$. Thus, at $K>0$ the proof remains analogous while paying attention just to the “tilded” Hilbert-space subspaces. $\square$

In the light of the closeness of parallels between the $K=0$ and $K > 0$ EP-related scenarios we leave the last-step $K>0$ upgrade of the construction of the admissible classes of observables (24) to interested readers. For compensation let us add here that in general, the variable parameters in metric (36) may be chosen $\alpha$-dependent, $c_n=c_n(\alpha )$. Fortunately, in the light of an appropriate upgrade of Lemma 5 it is clear that this would only imply an inessential modification of the physics described by the model.

## Discussion

The core of our present message may be seen in the not quite expected fact that the spiked and nonhermitian harmonic-oscillator Hamiltonian (3) offers a truly exceptional sample of a consequent application of the Bender-inspired, ${{\mathcal {PT}}}$-symmetry-based reformulation (we called it “three-Hilbert-space formulation”) of quantum mechanics of unitary systems. In this sense and in the light of the above-mentioned serious mathematical difficulties encountered during the study of the (non-spiked) imaginary cubic anharmonic oscillator the present, exactly solvable model could be assigned an important role of a new benchmark in the ${{\mathcal {PT}}}$-symmetric quantum theory.

In the language of mathematics such a upgrade of the status of the spiked harmonic-oscillator model can be perceived as a consequence of the existence of closed formulae (21) $+$ (23) and (35) $+$ (36). Near an arbitrary exceptional point of the system they describe the *complete*  set of the metric operators and, hence, they determine *all*  of the eligible, Hamiltonian-dependent physical Hilbert spaces ${{{\mathcal {H}}}}={{{\mathcal {H}}}}(H)$. This is precisely the situation in which one encounters an unrestricted possibility of an exhaustive “numbering” of the physical Hilbert spaces ${{{\mathcal {H}}}}(H)$ by the sets of parameters $\{b_n\}$ or $\{c_n\}$.

From the perspective of physics one can speak about the *complete* menu of the unitary quantum systems possessing the standard probabilistic interpretation and compatible with our preselected Hamiltonian *H*. In a way explained in^26^ (cf. also^6,34^ for some elementary illustrative examples), the ambiguity of this menu of spaces can subsequently be restricted (or even completely suppressed) by means of taking some other candidates $\Lambda$ for the observables into consideration.

In an opposite direction, in a way proposed in^35^) one could also recall the menu of metrics, pick up one of them, $\Theta _0$, and demand that any element $\Lambda _0$ of the class of eligible observables must satisfy the constraint

37 $$\begin{aligned} \Lambda _0^\dagger \,\Theta _0 =\Theta _0\,\Lambda _0\,. \end{aligned}$$

This just means that the knowledge of the complete menu of metrics enables us to keep also all of the admissible operators representing observables self-adjoint in our preselected Hilbert space ${{{\mathcal {H}}}}_0$.

In the latter context let us emphasize, last but not least, that the full generality of our present results concerning the special spiked-oscillator model opens also a truly remarkable possibility of studying an interplay between the influence of changes of the separate parameters. This could make the phenomenological interpretation of our apparently not too complicated model extremely flexible. *Pars pro toto * let us mention that one of the consequences of this flexibility (with a detailed analysis already lying far beyond the scope of our present short paper) might be sought near the reality-of-levels preserving unavoided-level-crossing interfaces with $\alpha ^{(EP)}=1,2, \ldots$. Indeed, at these boundary points we could admit *discontinuous*  jumps in the matrix elements $c_n=c_n(\delta )$ of the metric at $\delta =0$. Every such a jump (reflecting the “punched”, two-sided nature of the theoretically admissible diagonalizable-Hamiltonian vicinity of the EPs) would have to be interpreted as a genuine quantum catastrophe *alias*, in the terminology of Refs.^36,37^, a quantum phase transition of the second kind.

## Data Availability

No datasets were generated or analysed during the current study.
